# The striking mimics between COVID-19 and malaria: A review

**DOI:** 10.3389/fimmu.2022.957913

**Published:** 2022-08-23

**Authors:** Emadeldin Hassan E. Konozy, Makarim El-fadil M. Osman, George Ghartey-Kwansah, Hind Mohamed Abushama

**Affiliations:** ^1^ Department of Biotechnology, Africa City of Technology, Khartoum, Sudan; ^2^ Department of Zoology, Faculty of Science, University of Khartoum, Khartoum, Sudan; ^3^ Department of Biomedical Sciences, School of Allied Health Sciences, College of Health and Allied Sciences, University of Cape Coast, Cape Coast, Ghana

**Keywords:** *Plasmodium*, SARS-CoV-2, MIMICS, symptoms, pathological, immunological, molecular, ACE2

## Abstract

**Objectives:**

COVID-19 is a transmissible illness triggered by severe acute respiratory syndrome coronavirus 2 (SARS-CoV-2). Since its onset in late 2019 in Wuhan city of China, it continues to spread universally, leading to an ongoing pandemic that shattered all efforts to restrain it. On the other hand, in Africa, the COVID-19 infection may be influenced by malaria coinfection. Hence, in this review article, we aimed to give a comprehensive account of the similarities between COVID-19 and malaria in terms of symptoms, clinical, immunological, and molecular perspectives.

**Methodology:**

In this article, we reviewed over 50 research papers to highlight the multilayered similarities between COVID-19 and malaria infections that might influence the ontology of COVID-19.

**Results:**

Despite the poor health and fragile medical system of many sub-Saharan African countries, they persisted with a statistically significantly low number of COVID-19 cases. This was attributed to many factors such as the young population age, the warm weather, the lack of proper diagnosis, previous infection with malaria, the use of antimalarial drugs, etc. Additionally, population genetics appears to play a significant role in shaping the COVID-19 dynamics. This is evident as recent genomic screening analyses of the angiotensin-converting enzyme 2 (ACE2) and malaria-associated-variants identified 6 candidate genes that might play a role in malaria and COVID-19 incidence and severity. Moreover, the clinical and pathological resemblances between the two diseases have made considerable confusion in the diagnosis and thereafter curb the disease in Africa. Therefore, possible similarities between the diseases in regards to the clinical, pathological, immunological, and genetical ascription were discussed.

**Conclusion:**

Understanding the dynamics of COVID-19 infection in Sub-Saharan Africa and how it is shaped by another endemic disease like malaria can provide insights into how to tailor a successful diagnostic, intervention, and control plans that lower both disease morbidity and mortality.

## 1 Introduction

The Severe acute respiratory syndrome associated with the coronavirus (SARS-CoV-2) pandemic originated in Wuhan City, China at the end of 2019 (1) from there it extended to every corner of the globe leaving almost 4 million dead and more than 170 million infected (2). The central part of Africa, the Sub-Saharan zone, has a fragile and brittle health system and suffers for decades from malaria endemicity. A disease caused by the protozoa *Plasmodium*, species *falciparum* which is transmitted by the female *Anopheles* mosquito (3) and is accountable for the major death and cases in this stretch (4). Though the entire world has suffered a strong and continuous waves of the COVID-19 pandemic, Sub-Saharan Africa which extends from Sudan to Mauritania, persisted to the surprise of many scientists and researchers with the low COVID-19 occurrences and mortality rate as compared to the rest of the world (5). According to the recent WHO data on COVID-19 morbidity rates, the African region reported the least number of COVID-19 confirmed cases as of 25^th^ July, 2022 is (9,187,634) followed by the Eastern Mediterranean region (22,490,905), and highest numbers reported for the European region (238,567,709) (2). Thus far, there is no standardized protocol for the management of COVID-19 infection, nevertheless, several regimens including antimalarial, antiviral and immune system strengthening medications have been prescribed (6). Malaria and COVID-19 can have similar clinical presentations such as but are not limited to fever, backache, fatigue, shortness of breath, diarrhea, headache, stomach cramp, muscle pain, etc. (7). Additionally, several other common presentations at the clinical, pathological, and immunedeterminants levels have been illustrated. These common symptoms make the diagnosis challenging in places where proper health access and facilities are scarce, and diagnosis does not involve high throughput PCR testing used worldwide (8). Again, due to scarce facilities and large number of cases in most hospitals in Sub-Saharan Africa, empirical treatments are often given without laboratory testing (diagnosis). Thus, malaria cases might be misdiagnosed as COVID-19 or *vice versa* in both malaria-endemic and malaria-free zones (9), though the concomitant infection was also reported (10). Despite the African continent’s suffering from a poor and fragile healthcare systems, the documented morbidity and mortality due to COVID-19 remains puzzling (11). Many hypotheses and postulations such as the low testing numbers, the demographic factors, the climatic role and the travel dynamics between Africa and the rest of the world were put forward to explain the delay of COVID-19 manifestation and explain the low incidence in African countries with high burden and high incidence of malaria (12–14).

## 2 Genetic susceptibility/protection to malaria and COVID-19 infection

Genetic factors are among the most influential elements in susceptibility/resistance, progression, and outcome of a disease. That is, the genetically determined protection against an infectious disease of an individual is the reflection of inherently determining susceptibility to a life-threatening disease (15). The percentage of natural resistance to SARS-CoV-2 infection by humans is not known, however, a significant proportion of candidate genes possibly involved in the resistance to SARS-CoV-2 infection have emerged (15). Also, many polymorphisms have been selected by direct genetic pressure of *Plasmodium* in endemic areas (16). Shang and colleagues have recently suggested that COVID-19 arises due to a comparable interplay between viral spike proteins and angiotensin-converting enzyme 2 (ACE2) indicating the important function of spike protein in connection with the attachment and entry of the virus (17) and this has been reiterated by Sachdeva et al., (18). A current genomic study screened the ACE2 and genetic variants associated with malaria and found 6 genes that are probable predictors for key resistance or otherwise to COVID-19 and malaria incidence and severity (19). Included in the genetic variants are Glycophorin B, Interferon Regulatory Factor 1, Adenosine A2b Receptor, and Fc Fragment of IgG Receptor IIa. Interestingly, blood group O and ACE2 receptor expression were also part of the genetic variants identified (20). Polymorphisms in the ABO blood gene are suggested to contribute to the disparity in susceptibility of humans to microbial infections, SARS-CoV-2 inclusive (21). Although earlier reports on the effect of blood group on COVID-19 severity were varying, a recent meta-analysis of nearly 50,000 individuals from 46 studies confirmed its impact on susceptibility to infection. However, the protective role of the O allele is small, with an odds ratio of about 0.90. (15).

As indicated earlier, the ACE2 receptor has been suggested to be the key receptor for the entry of SARS-CoV-2 into the human body (19, 22). Angiotensin II (Ang-II) is the key player in this protection, it acts as a co-stimulator for CD4+ and CD8+ T cells activation, migration, differentiation, and cytokine production (23). It is regulated by the counteraction of both ACE1 and ACE2. Earlier reports indicated that the development of *P. gallinaceum* in birds is suppressed by ANG II. This shows the crucial role of Ang II in *Plasmodium* growth irrespective of the type of host. Thus, ANG II has the propensity to suppress sporozoite growth in the salivary gland of mosquitoes through disturbance of the parasite membrane (24). That is, *Plasmodium* invades red blood cells (RBCs) through ACE2 receptors located on the RBC surface. The ANG II interacts with the ACE2 receptor which converts it to Ang (1-7) (25). Angiotensin-(1–7) binds to its definite receptor MAS which is known to be expressed in the human erythrocyte membrane. A signalling pathway is then initiated to inhibit protein kinase-A (PKA) activity. Decreased activity of PKA reduces merozoite’s invasion of the erythrocytes (26).

**Figure 1:**
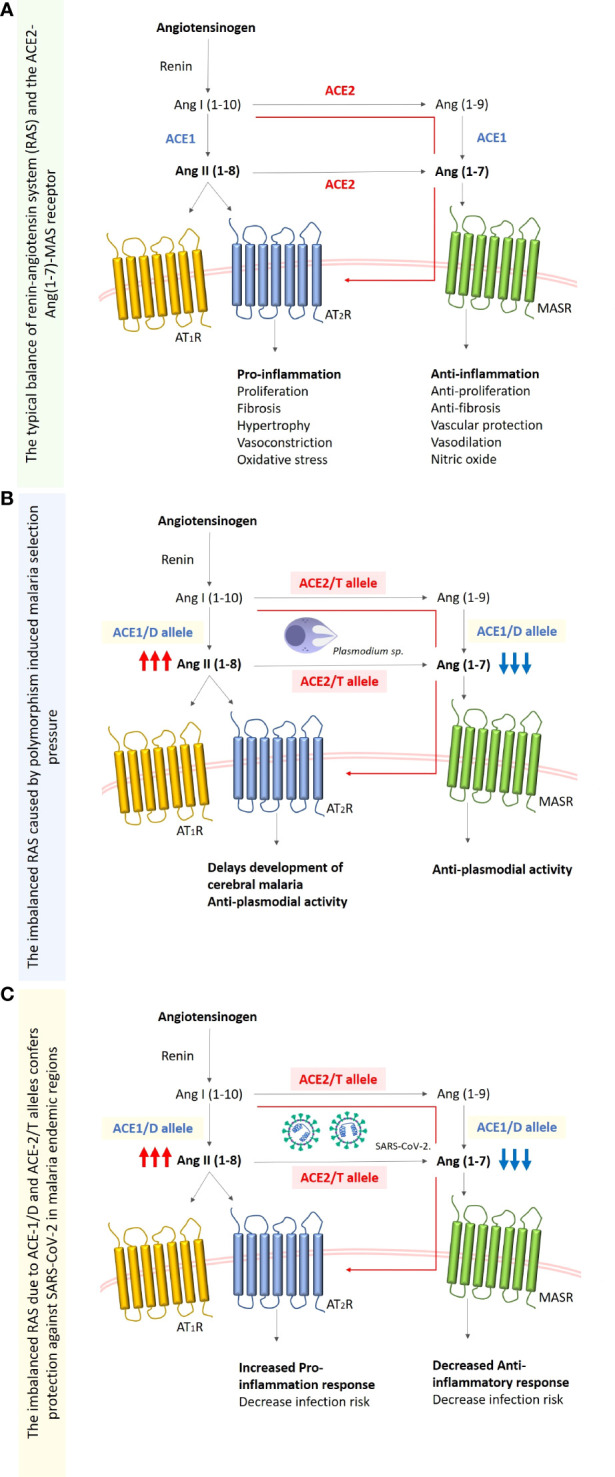
Simplified schematic diagram of the renin-angiotensinogen system (RAS). **(A)** Typical balanced RAS system. Renin claves angiotensinogen to angiotensin-I (Ang-I (1-10)) which is then converted to angiotensin-II (Ang-II (1-8)) by angiotensin-converting enzyme (ACE), the Ang-I is also converted to Ang (1-9) by angiotensin-converting enzyme-2 (ACE2), this enzyme also balances the excessive concentration of Ang-II by converting it to Ang (1-7). **(B)** The imbalanced RAS system caused by the chronic malaria exposure, and the subsequent polymorphism selection of the ACE-1 D allele that increases the enzyme levels and ACE-2 T allele (rs2106809) which results in the decreased expression of ACE2. This will increase Ang-II concentration which leads to an increase in the proinflammatory response, and the decrease of Ang (1-7) and subsequently the decrease in anti-inflammatory response. **(C)** SARS-CoV-2 infection in the malaria-endemic region where D and/or T alleles are fixed in the population due to malaria, is characterized by decreased concentrations of ACE and ACE2 respectively. This result in a reduced number of ACE-2 receptors required for viral entry, and also an increased pro-inflammatory response due to the increased concentrations of Ang-II (1-8).

There are evidences of association between hypertension and protection against cerebral malaria through polymorphism within the renin-angiotensin system (RAS) proteolytic cascade (27). Interestingly, since genetic polymorphism leads to a reduced expression of ACE2 by changing the concentration of ACE 1 (D alleles) in both infections, part of the correlation between SARS-CoV-2 and *Plasmodium* spp. can be seen by considering the ACE2 levels in populations with ACE1 (D allele) (28). Specifically, this polymorphism is linked with the variation of either bound-forms or circulatory ACE (19). In situations when the D allele dominates, this is associated with a reduced ACE2 receptor expression and might offer protection against COVID-19. Another report showed that the occurrence of D-allele ACE1/D polymorphism results in the production of ANG II and further mild malaria. Although the D-allele distribution varies across the globe (29), Sub-Saharan-Africans have been demonstrated to have higher levels of ANG II, resulted from ACE1/D polymorphism compared to Caucasians and various Asian populations (19, 30). Delanghe and colleagues argued that the log-transformed occurrence of SARS-CoV-2 infection was suggested to be indirectly connected to ACE D allele frequency and about 38% of the variability in prevalence can be ascribed to the relative incidence of the ACE1 D-allele (31). Furthermore, a substitution polymorphism (C→T) located in the intron-1 of the ACE2 gene, results in a reduction of the ACE2 expression; thereafter increasing the level of Ang-II (32). This may suggest that people with hypertension associated with ACE2 (C→T) and ACE D/I polymorphisms are protected against malaria and COVID-19 too due to the reduced level of ACE2 expression (33) (**Figure 1**).

Nonetheless, other mutations/polymorphisms related to protection against malaria are likely to increase the susceptibility and/or severity of SARS-CoV-2 infection. Glucose-6-phosphate dehydrogenase (G6PD) deficiency which is evolved by natural selection against malaria in Africa and Asia, can provide possible protection by combating parasite growth inside the RBCs or by inducing phagocytosis of the infected cells at the early stages of the blood infection cycle (34). G6PD is indirectly involved in regulating the innate proinflammatory-prooxidant and adaptive anti-inflammatory-antioxidant responses during microbial infection. This is taking place through the production of the cofactor NADH during glucose metabolism which is required for balancing both responses. Deficiency in G6PD expression in SARS-CoV-2 infected individuals results in imbalance of both immunological mechanisms, henceforth affecting the severe consequences of the disease (35). Another example of malaria protection is the null mutation of the Duffy antigen receptor for chemokine “DARC” which predominantly occur in Western and South-Western sub-Saharan African population and shields against *P. vivax* erythrocytes invasion (36). It was anticipated that the augmented ventilator-dependent organ failure and mortality risk among hospitalized African-American sub-population was owing to their erythroid Duffy null high frequency (37). SARS-CoV-2 patients with Duffy null mutation experience COVID-19-related pneumonitis due to the increased migration of leukocytes into the lung under the influence of the pro-inflammatory cytokines (38). Clinical evidence also points to the increased levels of tumor necrosis factor-alpha (TNF-α) in COVID-19 patients which is accompanied by the raised levels of other cytokines that lead to aggravated immune response (39). Yet, in certain areas of Africa, multiple types of functional mutations at the promoter of the TNF-α gene are retained over time through parasitic selection pressure. These mutations are shown to be responsible for the increased folds of expression of the protein which eventually contribute to the protection against *P. falciparum* and the elevated severity of COVID-19 disease (40).

## 3 Immunological similarities between malaria and COVID-19

The previous exposure to infectious diseases such as malaria might be the most convincing reason which leads to a sort of trained immunity to endemic pathogens which might explain the lower proportion of severe COVID-19 cases in many African countries even with fragile health care systems. For example, innate immune activation by different species of *Plasmodium* spp. would prime a more robust initial innate immune response to COVID-19 and could therefore protect against severe cases. This section of the paper reviews and attempts to associate the possible relationship between malaria and coronavirus immunity.

### 3.1 The innate immune response

Malaria is a powerful immunomodulator of adaptive immunity (41), and has been shown to also activate immunological memory in innate immunity similar to adaptive immunity. This immunological memory is capable of establishing an effective immune response against following infections and is also capable of providing cross-protection (42). The innate immune response against malaria involves major components such as natural killer (NK) cells, monocyte, macrophages, proinflammatory, and anti-inflammatory cytokines (41, 43). Accordingly, people living in malaria-endemic areas might be able to develop trained immunity and mount cross-protection which could be used against other pathogens like SARS-CoV-2 (44). The response is usually swift and characterized by the production of cytokines effective against subsequent infections.

Malaria infection starts from the liver stage which is clinically asymptomatic as the number of infected hepatocytes is relatively low (45). Consequently, this small number of infected hepatocytes tolerate parasite growth and generate a relatively high number of merozoites that are released into the bloodstream, initiating blood-stage infection. Parasite molecules are detected on the surface of infected erythrocytes through pattern recognition receptors (PRRs), mainly Toll-like receptors (TLRs), expressed by professional antigen-presenting cells (46, 47). These induce powerful innate responses that contribute to the detection, engulfment, and destruction of large numbers of infected erythrocytes, mainly in the spleen and liver (48, 49). In the case of SARS-CoV infection, after successful entry to the cell, the virus encodes different proteins that interact with downstream signaling PRR molecules present on immune cells, primarily the TLRs 3, 7, and 8, and with the JAK-STAT pathway (50). The host immune system then distinguishes the entire virus or its outer epitopes, triggering the innate or adaptive immune response. Recent Studies have provided evidence for the role of TLRs especially TLR4 in the induction of the host immune response against malaria and COVID-19. TLR4 is present mainly at the cell surface where it can detect viral proteins before they enter the cell and also in endosomes when its alternative signaling pathway is activated. It activates the production of type I interferons and proinflammatory cytokines to fight infection and has been suggested to be a promising therapeutic target (51, 52).

#### 3.1.1 The role of pro and anti-inflammatory cytokines in disease severity

A storm of pro-inflammatory cytokines including IL-1 and IL-6 and IFN-γ are produced in early malaria infection due to the direct destruction of parasitized RBCs by antigen-presenting cells such as macrophages, dendritic cells, and NK. This induces inflammation to restrict parasite growth (41, 53). IL-17 is a pro-inflammatory cytokine that has been shown to protect against extracellular bacteria and viruses (54). However, in *P. falciparum* infection, the secretion level of IL-17 was positively related to the severity and multiple-organ dysfunction including kidney failure (55).

In antiviral immunity, the production of pro-inflammatory cytokines like IFN-γ and TNF by NK cells play an important role during early infection. Evidence has been provided from *in vitro* study that NK cells display an anti-SARS-CoV-2 activity and also limit tissue fibrosis (56). However, NK cells from patients with COVID-19 were found to display a dysfunctional status similar to tumor-associated NK cells, which might compromise their anti-SARS-CoV-2 activity and potential antifibrotic activity, especially in individuals with severe disease (57).

These pro-inflammatory cytokines in both diseases must be regulated by anti-inflammatory ones because they can lead to severe disease and death when unregulated. The IFN-γ has been linked with resistance to malaria infection and to reinfection in children (43, 58, 59). Interferon-alpha and beta (IFN-α and IFN-β), collectively referred to as type-1 interferon (IFN-1) also play an important role in malaria immunity. The IFN-1 regulates the pro-inflammatory function of IFN-γ, thereby preventing an unbridled inflammatory response that can lead to severe disease (60). The dual role of IFN signalling in human malaria has been demonstrated. That is, increased amounts of IFN-1 improve anti-parasite responses by increasing IFNAR1 signalling in the early stages of infection, while exacerbated IFN-1/IFNAR1 signalling later in infection raises vulnerability to severe infection (61). The role of the host’s innate immune system is weakened throughout SARS-CoV infection by their non-structural proteins, which disturb the overall cytokine secretion (62). Viral innate immune cells are efficient in producing interferons that are involved in obstructing cell proliferation, apoptosis, immunomodulation, and reducing anti-inflammatory cytokines (63). Plasma obtained from patients with acute COVID-19 at 2- or 3 weeks post-infection, exhibited low production in IFN-α concentration to almost baseline. There was a further significant decrease in IFN-γ and TNF-α secretion and degranulation by NK cells, demonstrating that factors other than IFN-α exist in the later phase. For instance, the level of the proinflammatory cytokine IL-6 was decreased in the plasma of patients with acute COVID-19 from the first to the third week. The decreased IL-6 level overturns IFN-γ production in NK cells *in vitro* and possibly affects the normal function of NK cells in patients with severe illness. The impacts of IFN signaling on COVID-19 pathology are multiple, with both protective and harmful effects being documented. A multi-omics biosignature associated with varying levels of 12 different type I, II, and III IFNs has been defined in systemic IFN signalling in hospitalized COVID-19 patients (64). Production of cytokines and chemokines, which induce the immune cells in the lungs was increased, hence resulting in acute respiratory distress syndrome (ARDS), which is fatal for patients (65). Indicative cytokines in severely ill COVID-19 patients are characterized by boosted expression of IL-6, TNF-α, IL-17 macrophage inflammatory proteins 1-α (MIP-1α), MCP3, GM-CSF, IL-2, and IP-10, in addition to the elevated chemokines (IP-10, CCL2/MCP1, CXCL1, CXCL5) (58, 66). This further enhances the exhaustion of effector T cells and decreases the immune response against the virus (67). An apparent rise of IL-17 in patients has been observed in comparison to the control patients with a lowered death rate and decreased immune-related lung injury (68).

The robust pro-inflammatory cytokine production levels (hyperresponsiveness) are maintained in malaria-endemic areas due to repeated infection. Immunotolerance (hypo-responsiveness) is built by immunomodulatory molecules mainly IL-10 and TGF-β that activates the anti-inflammatory cascade reaction, tempers hyperinflammation and prevents tissue damage and might trigger a chronic immune reaction (69–71).

This tolerance guarantees the limitation of the destructive inflammatory effects that is accountable for severe malaria, common in persons without malaria immunity (72). The hyperresponsiveness and tolerance triggered by malaria could also be cross-protective from following infections by other endemic microbe and beneficial to tackling the disease severity. Therefore, it might be the reason people living in malaria-endemic areas are protected against the severe inflammatory response of COVID-19 (73). A more or less similar effect has been reported in other infections. Specifically, pre-existing T cell immunity to common cold coronaviruses can establish the response to SARS-CoV-2 (74).

### 3.2 The adaptive immune response

The innate response is followed by activation of the CD4 cells with subsequent clonal expansion, producing Th1 and Th2 in malaria infection that result in cell-mediated and antibody-mediated immunities, respectively (41, 53). The Th2 CD4 cells activate B cells to produce antibodies, especially immunoglobin G (IgG), which is the major antibody that triggers this cascade of immune reactions (75). In some malaria-endemic areas, high levels of circulating IgG have been associated with minimizing malaria risks (53, 76, 77)

During SARS-CoV-2 infection, humoral immunity involves a strong CD4+ T-cell response (74) and the characteristic production of IgG, IgA, and IgM (68). At the beginning of SARS-CoV infection, B cells trigger an early reaction against the Nucleocapsid (N) protein, while antibodies against the Spike (S) protein could be detected after 4 days to a week from the presence of initial symptoms (78, 79). Immunoglobin G appears to be pivotal in the recovery of persons with COVID-19 (80). Nevertheless, it has been observed that weak responders for IgG antibodies had higher viral clearance than strong responders. This observation may probably suggest that a vigorous antibody response leads to disease severity while a feeble response is associated with the elimination of the virus, However, this study tested IgG against SARS-CoV-2 nucleocapsid protein but not spike proteins in a few number of patents which might not reflect the true situation in COVID-19 patients (81).

Antibodies produced in malaria infection target free circulating merozoites and parasitized RBCs, preventing merozoites invasion of RBCs and the cytoadherence of parasitized RBCs on the endothelium (41). This attachment of the antibodies to the merozoites and parasitized RBCs could result in opsonization and subsequent phagocytosis, complement-mediated cell destruction or antibody-dependent cell-mediated destruction of parasitized RBCs (41, 53).

The immune cells and their products (cytokines and antibodies) produced during malaria infection, can remain for years as memory cells and as circulating cytokines and antibodies if exposed to repeated infections (41, 43, 53). Moreover, NK cells have been considered as part of both innate and adaptive immunity. Previous studies suggest their capacity to maintain memory and derive a memory-like response (43, 82).

During COVID-19 infection, the concurrent presence of robust SARS-CoV-2 anti-spike antibodies and memory cell responses specific for SARS-CoV-2 peptides involving NK cells through characterized secretion of IFN –γ have been demonstrated in patients who recovered from COVID-19 up to 7 months post-infection (83). The observed dynamics of the humoral and adaptive immune response identified in previous studies reported that anti-spike IgG remained stable up to 6 months after diagnosis (84). Effective vaccine or T cell epitopes could be used to target a particular population for rapid viral clearance. COVID-19 subjects have reduced regulatory T cell populations and memory T cells, which may aggravate the inflammatory response leading to cytokine storm and hence, enhance tissue damage and organ failure (85).

**Figure 2:**
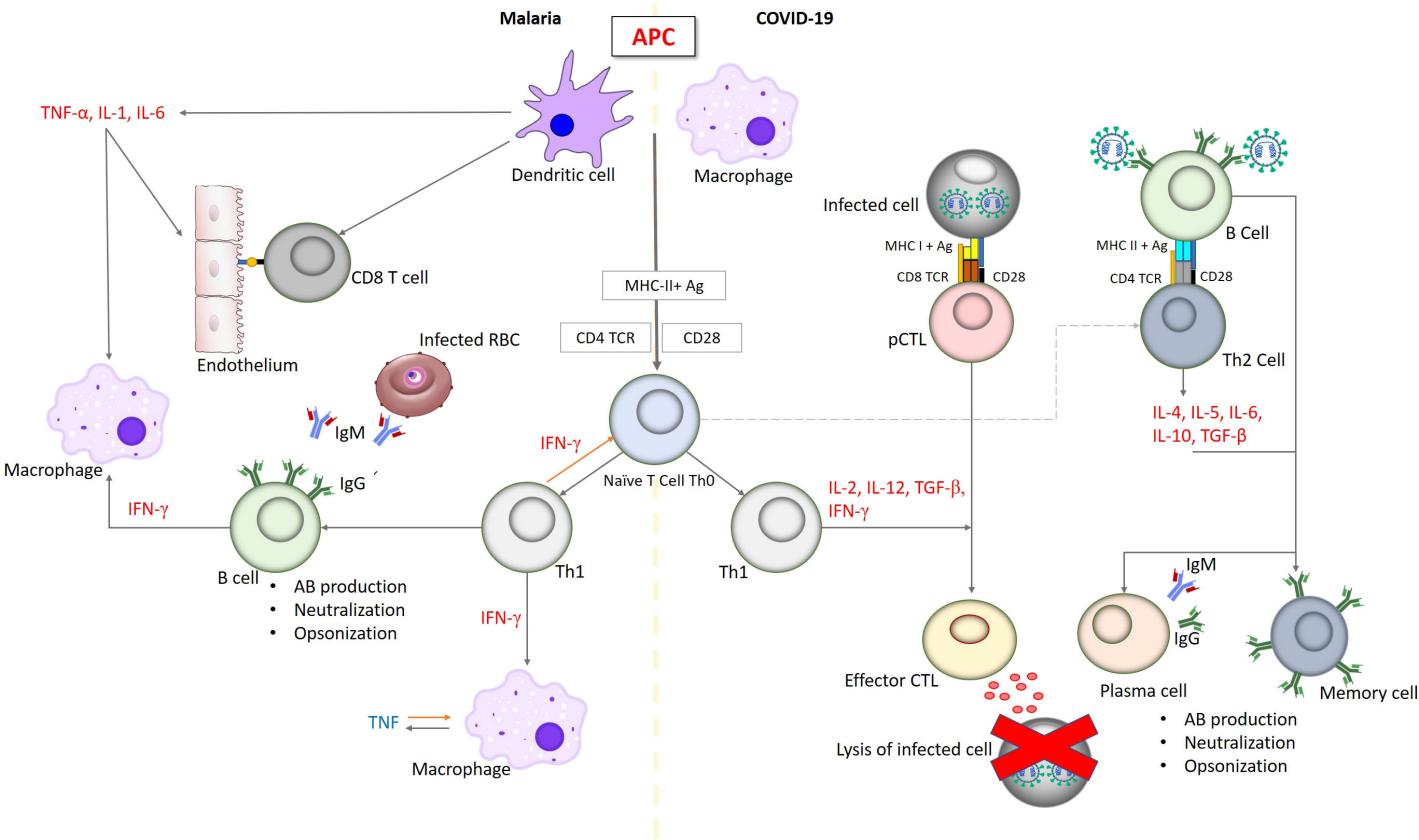
Simplified outline of immune responses in malaria and infections. Innate immune response in both malaria and COVID-19 begins with involving antigen-presenting cells, macrophages, dendritic cells and natural killer cells resulting in a sharp blowout of pro-inflammatory cytokines including IL-6, TNF and IFN promoted to inhibit pathogen proliferation. These pro-inflammatory cytokines are regulated by anti-inflammatory cytokines such as IL-10 and TGF-b to reduce the severity and increase the ability of tissues to tolerate inflammatory damage. CD8+ TC lymphocytes, including memory cells, recognize pathogen epitopes and cross-reactive epitopes from related pathogens leading to the effective killing. Antibodies are produced to prevent the pathogen from invasion of infected cells and the cytoadherence which could result in opsonization and subsequent phagocytosis. IgG is the major antibody that prompts this cascade of immune reactions.

Overall, acute infections of malaria and SARS-CoV-2 resulted in a comparably elevated activation and altered differentiation status of the CD8+ and CD4+ T cell populations. The outcome of both malaria and COVID-19 is thought to be a consequence of the balance between co-activation and co-inhibition of T cells. The potential immune response similarities between malaria and covid-19 were summarized in **Table 1**, and **Figure 2**.

**Table 1:** Potential similarities in innate and adaptive immune mechanisms against malaria and COVID-19.

	Malaria	References	COVID-19	References
**Innate immunity**	Pattern recognition receptors (e.g., TLRs) expressed by immune cells and STAT1/STAT2-IRF9 pathway contribute to recognition and engulfment of infected erythrocytes	(46, 47)	Pattern recognition receptors (e.g., TLRs) present on immune cells and the JAK-STAT pathway are the first to identify the virus	(50, 86)
	Innate immune response begins with involving neutrophils, macrophages, dendritic cells and natural killer cells resulting in a sharp blowout of pro-inflammatory cytokines including (IFN) that induce inflammation to inhibit parasite growth	(41, 53)	Viral innate immune cells are efficient in producing IFNs involved in blocking cell proliferation, apoptosis, and immunomodulation.	(63, 87)
	These pro-inflammatory cytokines are regulated by anti-inflammatory cytokines because they can lead to severe malaria and death when unregulated	(41, 53)	Secretion of cytokines and chemokines, which attract the immune cells to the lungs, was increased, hence causing ARDS, which is fatal to severely ill individuals	(65)
	Interferon-alpha and beta regulate the pro-inflammatory function of interferon-gamma, thereby preventing chaotic inflammatory response that can lead to severe disease	(60)	Secretion of cytokines and chemokines, which attract the immune cells to the lungs, was increased, hence causing ARDS, which is fatal to severely ill individuals	(88)
	NK cells are involved in the direct destruction of parasitized RBCs and the production of pro-inflammatory cytokines early in malaria infection	(43, 53)	NK cells display an anti-SARS-CoV-2 activity and showed to limit tissue fibrosis during early infection	(89)
	The dual role of IFN signalling in human malaria where increased amounts of IFN-1 improve anti-parasite responses by increasing IFNAR1 signalling in the early stages of infection while exacerbated IFN-1/IFNAR1 signalling later in infection increases vulnerability to severe disease	(61)	Signalling by interferon (IFN) affects COVID-19 pathology in both protective and harmful ways. A multi-omics biosignature associated with varying levels of 12 different type I, II, and III IFNs has been defined in a systemic IFN signalling in hospitalized COVID-19 patients	(64)
**Adaptive immunity**	Activation of CD4 cells, resulting in cell-mediated and antibody-mediated immunity, respectively	(41)	Humoral response against SARS-CoV-2 involves a strong CD4^+^ T-cell response and the crucial production of IgG, IgA and IgM.	(74)
	Antibodies produced prevent merozoites invasion of RBCs and the cytoadherence of parasitized RBCs on the endothelium which could result in opsonization and subsequent phagocytosis, complement-mediated cell destruction or antibody-dependent cell-mediated destruction of parasitized RBCs	(41)	Antibody-Dependent Enhancement (ADE) occurs through non-neutralizing antibody enhanced the mechanism of viral entry that results in atypical activation of immune cells	(90)
	Immunoglobin G is the major antibody that prompts this cascade of immune reactions. In some malaria-endemic areas, high levels of circulating immunoglobin G have been associated with lower malaria risks	(76, 77)	IgG antibodies had higher viral clearance. A vigorous antibody response leads to disease severity while a weak response is associated with the elimination of the virus	(78)
	CD8+ T_C_ lymphocytes are activated through antigen cross-presentation by DCs. IFN-γ-producing CD8^+^ T cells operate on inflammation and cytotoxicity (perforin and granzyme B mediated) functions	(91)	CD8+ T_C_ lymphocytes, including memory cells, recognize SARS-CoV-2 epitopes and cross-reactive epitopes from related coronaviruses. Cytotoxicity to virus-infected cells mediated through granzyme and perforin	(92)

It could be highly speculated that malaria activates both innate and adaptive immunity to subsequent infections, but it seems the effect of innate immunity is more prominent. The main modulators of this scenario are natural killer cells and interferons resulting in protection against severe malaria (93). Individuals exposed to malaria infection and can develop immunity may have circulating primed innate cells like natural killer cells and monocytes and have protective antibodies, mainly IgG. Those might be capable of mounting a prompt, fast and successful response against COVID-19, mainly as production of effective cytokines and antibodies without passing the condition to a severe case.

## 4 Cellular invasion routes similarities between malaria and COVID-19

Entry of SARS-CoV-2 into human cells *via* ACE-2 is globally accepted. Erythroid Progenitors (EPs) infection by the virus through their highly expressed surface receptor ACE-2 accentuates this infection during erythropoiesis stages (94). Additionally, in the early days of EPs proliferation, the expression of receptors like CD147 and CD26 was also noted (95). SARS-CoV-2 infection through CD147, which is an erythrocyte surface receptor, targeting nucleated bone marrow and young RBCs through endocytosis has been postulated by several researchers (5, 96, 97). In a recent publication, Zhou et al. had proven that a SARS-CoV-2 pseudovirus could successfully invade the *Vero E6* cells, nevertheless, this invasion was abrogated when a CD147 knockdown cell was used instead (98). It is noteworthy that CD147 (also known as Basigin or EMMPRIN) provides a route for *plasmodium* to invade RBC, a process that is mediated through the blood-stage merozoite surface antigen PfRH5 (99, 100). Using a biophysical model, the *Resonant Recognition* (RRM) which analyzes proteins while interacting with their target receptors, Cosic and his colleagues, have theoretically indicated a probable affinity attraction between SARS-CoV-2 S1 Spike protein and RBC band-3. Interestingly a similar interaction, however, with different RRM frequency was noticed, between RBC band-3 and merozoite surface protein PfRH5 of the *P. falciparum*. Due to the variabilities in the RRM frequencies, the authors suggested a different mechanism for RBC band-3 and merozoite interaction than that of merozoite-SAR-CoV-2 S1 spike protein (101). These studies collectively underline a plausible role of SARS-CoV-2 inside RBCs comparable to that of the blood-stage malaria. This would eventually lead to COVID-19 perceived clinical and pathological findings such as hemolytic anemia and thrombosis at the severe stages (102, 103). Complimentary to this, is a combined proteomic, metabolomics, and lipidomics study carried out on 29 COVID-19 patients in the United States. In this study, RBC was shown to exhibit a deformed cellular membrane, disturbed erythrocyte’s glycolytic pathway, and degraded N-terminal cytosolic domain of band 3. All of these findings were interpreted as a result of a possible invasion of SARS-CoV-2 to erythrocytes (104). Interestingly, in human erythrocytes, band-3 plays a paramount role as a receptor for the sialic acid-independent binding of *P. falciparum* merozoite protein 1 during the RBC infection by the parasite (105).

## 5 Bioinformatics similarities between malaria and COVID-19

An *in-silico* proteome search was performed by Adiguzel for the 5 malaria-causing parasite species (*P. falciparum, P. malariae, P. vivax, P. ovale, and P. knowlesi*) against the human proteome, and the resultant peptides similarity of more than 60% identity were selected and tested *in silico* for possible binding affinities to MHC class I. In this study, the author had shown that 5 peptides from *P. vivax* possessed high affinity towards HLA-A*24:02, whereas a single human peptide (YFYLFSLELF) was found to share homology with *P. falciparum* and would induce strong binding to the same HLA allele (106). Another study executed on *P. falciparum* predicted partial four SARS-CoV-2 immunodominant cross-reactive epitopes to the malaria parasite *P. falciparum* antigens’ TRAP and SSP-1 which bind to the HLA-A*02:01 alleles that are restricted to CD8^+^ T-lymphocytes response in malaria-endemic regions and speculated to curb the rapid dissemination of SARS-CoV-2 infection in these areas (5). Comparing the amino acid sequence of SARS-CoV-2 with that of Bat coronavirus (Bat-CoV) revealed an astonishing replacement of 7 amino acids motif (_439_NNLDSKV_445_) found in the SARS-CoV-2 spike protein and missing in that of Bat-CoV. Upon the database search, these 7 amino acids motif was found to share 100% similarity with *P. malariae* conserved membrane bound-surface protein. Performing a CD4 T-cell epitope analysis resulted in two epitopes with the core peptide motif (NNLDSKV) that have 97.72 and 98.60% immunogenicity and bind MHC-I alleles (HLA-A*24:02, HLA-A*02:01 and other alleles) (107, 108). While the preparation of this review was underway an international group headed by Prof. Bei, Yale University demonstrated that antibodies generated against malaria would interact with SARS-CoV-2 surface protein. The antibodies provoked by severe *Plasmodium* infection recognize the S1 subunit of the SARS-CoV-2 Spike glycoprotein. This interaction was mediated through the terminal sialic acid, removal (by glycosidase) of which abolished these interactions. Inversely, under *in vitro* conditions, the antibodies secreted against *P. falciparum* antigens did not neutralize the SARS-CoV-2. Based on these results authors concluded that previous malaria infection may not provide any protection against SARS-CoV-2 contagion (109). Antibody-antigen interaction is known to be influenced by many factors such as temperature, pH and ionic strength. False-positive results are noted under low ionic strength (110). However, since these authors’ experiment was merely performed under *in vitro* settings and they did not provide thorough experimental details it becomes hard to comment. In an interesting letter to the editor article, Panda and his colleagues suggested that α-(1,3)-galactose specific IgG and IgM produced excessively among individuals residing in malaria-endemic areas can cross-react with multiple epitope determinants of other pathogens, hence could also interact with SARS-CoV-2 glycoproteins (111). The largest SARS-CoV-2 accessory protein ORF3a which plays a critical role in viral replication attacks erythrocytes and dissociates heme (65). Another *in-silico* study revealed similar domains from SARS-CoV-2 and *Plasmodium* antigens that show comparable interactions with erythrocytes (112). An interesting result was put forward by Charon et al, in which an arenavirus-like sequence scientifically known as Matryoshka RNA virus 1 (MaRNAV-1) was detected by meta-transcriptotomics in the blood samples obtained from patients infected by *P. vivax*. Further analysis carried out by the authors confirmed the infection of the parasite by the Matryoshka RNA virus. Remarkably this viral association with the *Plasmodium* was exclusive to *vivax* but not detected with any other *Plasmodium* species (113). Investigating the possible antigenic sites in SARS CoV-2 spike glycoprotein receptor resulted in 9 potential sites. Seven of them had antigenic similarities with many pathogenic bacteria and interestingly two malaria parasites *P. falciparum* and *P. knowlesi* (114). These results together might highlight a possible evolutionary liaison between the two microbes and perhaps contribute to rationalizing the reported cross-immunity between *Plasmodium* and SARS-CoV-2 (5, 106).

## 6 Conclusion

Malaria is a life-threatening illness triggered by a parasite, the *Plasmodium*. This protozoan can influence its host genetically and define a population’s genetic makeup. It can also impact the host immune responses to co-infection with other pathogens. The ostensible astonishing similarities between COVID-19 and malaria urge further investigations into the immunology and genetics of these two microbes. Investigations on COVID-19 patients living in malaria-endemic areas to assess the status of adaptive immunity in comparison to healthy individuals will offer important immunological information in connection with natural killer cells, interferons (INF-γ and INF-I), and IgG. These immunological agents should then be evaluated among symptomatic and asymptomatic COVID-19 patients. Obtaining such data will shed light on the starring aspects of malaria-induced trained immunity and immune tolerance in COVID-19 patients. Bearing in mind the variation in exposure to *Plasmodium* in endemic areas as some individuals might fail to develop such immunity. Moreover, such investigations will not only pave the way to understanding COVID-19 infection and mechanism of action in malaria-endemic regions but also assist in designing and manufacturing an effective vaccine to combat long-lasting malaria.

## Author contributions

EK: Coined the idea, participated in writing and editing the manuscript. MO: Drew the figures and participated in writing and editing the manuscript. GG-K: Participated in writing and editing the manuscript. HA: Participated in writing and editing the manuscript. All authors contributed to the article and approved the submitted version.

## Conflict of interest

The authors declare that the research was conducted in the absence of any commercial or financial relationships that could be construed as a potential conflict of interest.

## Publisher’s note

All claims expressed in this article are solely those of the authors and do not necessarily represent those of their affiliated organizations, or those of the publisher, the editors and the reviewers. Any product that may be evaluated in this article, or claim that may be made by its manufacturer, is not guaranteed or endorsed by the publisher.
